# Src and Memory: A Study of Filial Imprinting and Predispositions in the Domestic Chick

**DOI:** 10.3389/fphys.2021.736999

**Published:** 2021-09-20

**Authors:** Maia Meparishvili, Lela Chitadze, Vincenzo Lagani, Brian McCabe, Revaz Solomonia

**Affiliations:** ^1^School of Natural Sciences and Medicine, Institute of Chemical Biology, Ilia State University, Tbilisi, Georgia; ^2^Department of Zoology, University of Cambridge, Cambridge, United Kingdom; ^3^I. Beritashvili Centre of Experimental Biomedicine, Tbilisi, Georgia

**Keywords:** learning, recognition memory, IMM, intermediate medial mesopallium, memory formation, early learning

## Abstract

Visual imprinting is a learning process whereby young animals come to prefer a visual stimulus after exposure to it (training). The available evidence indicates that the intermediate medial mesopallium (IMM) in the domestic chick forebrain is a site of memory formation during visual imprinting. We have studied the role of Src, an important non-receptor tyrosine kinase, in memory formation. Amounts of total Src (Total-Src) and its two phosphorylated forms, tyrosine-416 (activated, 416P-Src) and tyrosine-527 (inhibited, 527P-Src), were measured 1 and 24 h after training in the IMM and in a control brain region, the posterior pole of nidopallium (PPN). One hour after training, in the left IMM, we observed a positive correlation between the amount of 527P-Src and learning strength that was attributable to learning, and there was also a positive correlation between 416P-Src and learning strength that was attributable to a predisposition to learn readily. Twenty-four hours after training, the amount of Total-Src increased with learning strength in both the left and right IMM, and amount of 527P-Src increased with learning strength only in the left IMM; both correlations were attributable to learning. A further, negative, correlation between learning strength and 416P-Src/Total-Src in the left IMM reflected a predisposition to learn. No learning-related changes were found in the PPN control region. We suggest that there are two pools of Src; one of them in an active state and reflecting a predisposition to learn, and the second one in an inhibited condition, which increases as a result of learning. These two pools may represent two or more signaling pathways, namely, one pathway downstream of Src activated by tyrosine-416 phosphorylation and another upstream of Src, keeping the enzyme in an inactivated state via phosphorylation of tyrosine-527.

## Introduction

Src is a non-receptor protein tyrosine kinase that participates in a number of neuronal processes including neurotransmitter release, neurotransmitter receptor function, and synaptic plasticity. Autophosphorylation of tyrosine-416 in the activation loop of Src is thought to increase Src activity, whereas phosphorylation at tyrosine-527 by other kinases suppresses Src activity (Ohnishi et al., 2011). There is evidence that Src participates in learning and/or memory, possibly via phosphorylation of *N*-methyl-d-aspartate receptors, but its role is poorly understood. Overexpression of Src affects excitatory synaptic transmission in area CA3 of the mammalian hippocampus, and impairs fear memory (Yan et al., 2017). After spatial maze learning, upregulation of Src mRNA was observed in area CA3 (Zhao et al., 2000). Increased autophosphorylation of Src enhances hippocampal long-term potentiation and spatial memory (Wang et al., 2018). To clarify the role of Src in memory, it would be helpful to simultaneously measure its activated form phosphorylated at tyrosine-416 (416P-Src) and its inactive form phosphorylated at tyrosine-527 (527P-Src) in a vertebrate brain region where memory is encoded, and for a type of learning for which a graded measurement of memory strength is available.

Visual filial imprinting in the domestic chick is a type of learning having these favorable characteristics for memory research. Imprinting is a process whereby, when exposed to a salient visual stimulus (training), a chick typically approaches, learns the features of, and subsequently recognizes the stimulus. Memory strength may be measured in terms of behavioral preference, namely approach to the training stimulus relative to approach to a stimulus not previously seen (Bolhuis, 1991; Horn, 2004; McCabe, 2019). The intermediate medial mesopallium (IMM) is a chick forebrain region of crucial importance for visual imprinting, and the available evidence indicates that this region stores information about the imprinting stimulus (Horn, 1985, 2004). Criteria used to infer that a change following training is learning-related have been formulated (Horn and Johnson, 1989; McCabe, 2013; Solomonia and McCabe, 2015; Margvelani et al., 2018a) (see also Discussion).

The IMM resembles mammalian association cortex (Horn, 1985) and, from evidence based on the distribution of cholecystokinin mRNA expression, homology with mammalian neocortical layers 2 and 3 has been proposed (Atoji and Karim, 2014). The IMM is also important in passive-avoidance learning (Rose, 2000), the nearby anterior medial mesopallium in auditory imprinting (Bredenkötter and Braun, 1997), and the caudal medial mesopallium in memory of tutor song in songbirds (Gobes et al., 2010).

The left and the right IMM are involved in imprinting in different ways (Horn, 2004). Shortly after training, learning-related molecular changes occur on both the left and the right sides of the IMM, whereas ~24 h after training they are predominantly expressed in the left IMM. Learning-related changes also occur in the right IMM at 24 h, but in general are weaker than on the left side (Solomonia and McCabe, 2015).

Several proteins are changed in a learning-related manner following imprinting (reviewed in Solomonia and McCabe, 2015). The affected proteins include, among others, cell adhesion molecules, neurotransmitter receptors, protein kinases and their substrates, transcription factors, mitochondrial and membrane proteins, vesicle recycling components, and translation factors (McCabe and Horn, 1988, 1994; Sheu et al., 1993; Solomonia et al., 1997, 1998, 2000, 2003, 2005, 2008, 2011, 2013; Meparishvili et al., 2015; Margvelani et al., 2018a,b; Chitadze et al., 2020). Further results indicate that neurotransmitter release in the IMM is modulated with memory after imprinting (McCabe et al., 2001; Meredith et al., 2004).

Statistical analysis has indicated that certain biochemical quantities in the IMM that are correlated with the strength of learning are not the result of learning, but exist prior to training and reflect processes associated with a predisposition to learn well. Such processes thus give rise to rapid learning when training occurs. A microRNA with this property has been identified in the left IMM (Margvelani et al., 2018a).

In the present study, we have inquired whether the amounts of total Src, 416P-Src (putative activated form), and 527P-Src (putative inhibited form) are changed in a learning-related manner 1 h and 24 h after imprinting training. Four brain regions were analyzed, the left and the right IMM and two control forebrain regions, which are not involved in imprinting, the left and the right posterior pole of the nidopallium (PPN). We find a strong relation between the strength of learning and the level of 527P-Src at both times in the left IMM. In addition, there was evidence at both times implicating 416P-Src in the left IMM in a predisposition to learn readily. Twenty-four hours after training, Total-Src was dependent on strength of learning in both the left and the right IMM.

## Materials and Methods

### Behavioral Training and Testing

Fertile eggs (Cobb 500) were obtained from Sabudara farm, Tbilisi, Georgia. Nineteen batches of eggs were incubated and hatched in darkness, and the chicks were reared in isolation in darkness and trained for 1 h. Chicks from 10 batches were used in experiments in which measurements were made 1 h after the end of training; in the remaining nine batches measurements were made 24 h after the end of training. In each batch, there were up to three trained chicks and a control chick from the same hatch. At 22–28 h post-hatch, each chick to be trained was exposed in a running wheel (1 revolution = 94 cm) to a training stimulus (a cuboidal red box rotating about a vertical axis) for 1 h. The box contained a light surrounded by a red filter (Lee Filters 106 Primary Red); the largest pair of sides of the box (18 ×18 cm) were translucent and vertical and the remaining sides (18 ×9 cm) were black. During training, the stimulus was turned on for 50 s and then off for 10 s each minute. The maternal call (70–75 dB) of a hen was played while the stimulus was on, a procedure that accelerates imprinting to a visual stimulus (Smith and Bird, 1963). As a chick attempted to approach the training stimulus, it rotated the running wheel and revolutions of the wheel were counted to provide a measure of approach activity (“training approach”). A preference test without the maternal call was performed 10 min after training, in which each chick in a running wheel was shown sequentially the training stimulus and an alternative stimulus that the chick had not previously seen, in the order training/alternative/alternative/training. Each period of exposure during the test lasted 4 min, making a total of 8 min for each stimulus. The alternative stimulus was a right circular cylinder (height 18 cm and diameter 15 cm) with a translucent wall and vertical axis, rotating about this axis at 28 revolutions per minute. The cylinder contained a light surrounded by a blue filter (Lee filters HT 118 Brilliant Blue). See Horn (1998) for illustrations of the training and alternative stimuli. A preference score (approach to training stimulus during test × 100/total approach during test) measured the strength of imprinting (i.e., learning). A preference score of ~50 indicates poor learning, whereas a score of ~100 indicates strong learning. There are individual differences in the preference scores of chicks after a fixed period of training. This variation was used to determine whether the amount of protein was related to preference score and, by means of subsequent analysis (see below and Discussion), whether a change in protein amount was attributable to learning that occurred during training. Chicks were decapitated either 1 or 24 h after the end of training. Four tissue samples were removed from each chick, from the left and the right IMM and from the left and the right PPN, and immediately frozen on dry ice. Thus in each batch, there were four samples from each of up to four chicks (one untrained, up to three trained), yielding up to 16 samples in all. The locations of the IMM and PPN are shown in a previous publication (Solomonia et al., 2013). For details of IMM removal, see Davies et al. (1985) and for PPN removal see Solomonia et al. (1998). Samples were coded after collection, and all further procedures were conducted blind. All behavioral experiments were carried out at the I. Beritashvili Centre of Experimental Biomedicine and performed in compliance with its approved animal care guidelines. The number of animals used was found sufficient in previous studies to permit reliable detection of correlations between biochemical measures in the IMM and preference score.

### Sample Preparation, SDS Electrophoresis, and Western Blotting

Samples were rapidly homogenized in standard Tris-HCl buffer containing phosphatase inhibitor and protease inhibitor cocktails, and sodium dodecylsulphate (SDS) solution was added at a concentration of 5% and the mixture was at 95°C for 3 min. Protein concentrations were determined in quadruplicate using a micro bicinchoninic acid protein assay kit (Pierce). Aliquots containing 30 μg of protein in 30 μl were subjected to SDS gel electrophoresis and Western blotting (Meparishvili et al., 2015). After protein had been transferred onto nitrocellulose membranes, the membranes were stained with Ponceau S solution to confirm transfer and uniform gel loading. In each batch, samples were run in triplicate, where one filter was stained with polyclonal antibody raised against total Src protein (see below), the second one with antibody against 416P-Src (Abcam, ab4816), and the third one with antibody against 527P-Src (Abcam, ab4817).

Rabbit polyclonal antibodies against chicken Src (UNIPROT P00523) were produced using an epitope the 19-mer peptide RRSLEPPDSTHHGGFPASC (amino acid residues 15–32 of chicken Src), which contained a terminal cysteine for conjugation to a carrier protein. Antibodies were purified on an antigen-affinity column. This epitope does not coincide with known phosphorylation sites, and thus recognizes both phosphorylated and non-phosphorylated forms of Src. The specificity of antibody reaction was confirmed by adsorption of the control peptide.

Standard immunochemical procedures were performed using peroxidase-labeled secondary antibodies and SuperSignal West Pico Chemiluminescent Substrate (Pierce-Thermo Fisher Scientific, Waltham, Massachusetts, USA). Blots were then exposed with intensifying screens to X-ray films preflashed with Sensitize (Amersham; GE Healthcare Life Sciences, Little Chalfont, Buckinghamshire, UK). Optical density of protein bands was measured using LabWorks 4.0 software (Ultra-Violet Products Ltd., Cambridge, UK). Autoradiographs on each gel were calibrated using standard amounts of protein (15, 30, 45, and 60 μg total protein) obtained from homogenate fractions of the IMMs from a group of untrained chicks. For these standards, the optical densities of bands immunostained for the corresponding protein were linearly related to the amount of protein (Supplementary Figure S1). To obtain data for regression analysis the optical density of each band from each sample was divided by the optical density which, from the calibration of the same autoradiograph, corresponded to 30 μg of total protein in the standard (Meparishvili et al., 2015). This quantity is termed “relative amount of protein”.

### Statistical Analysis

**Figure 1:**
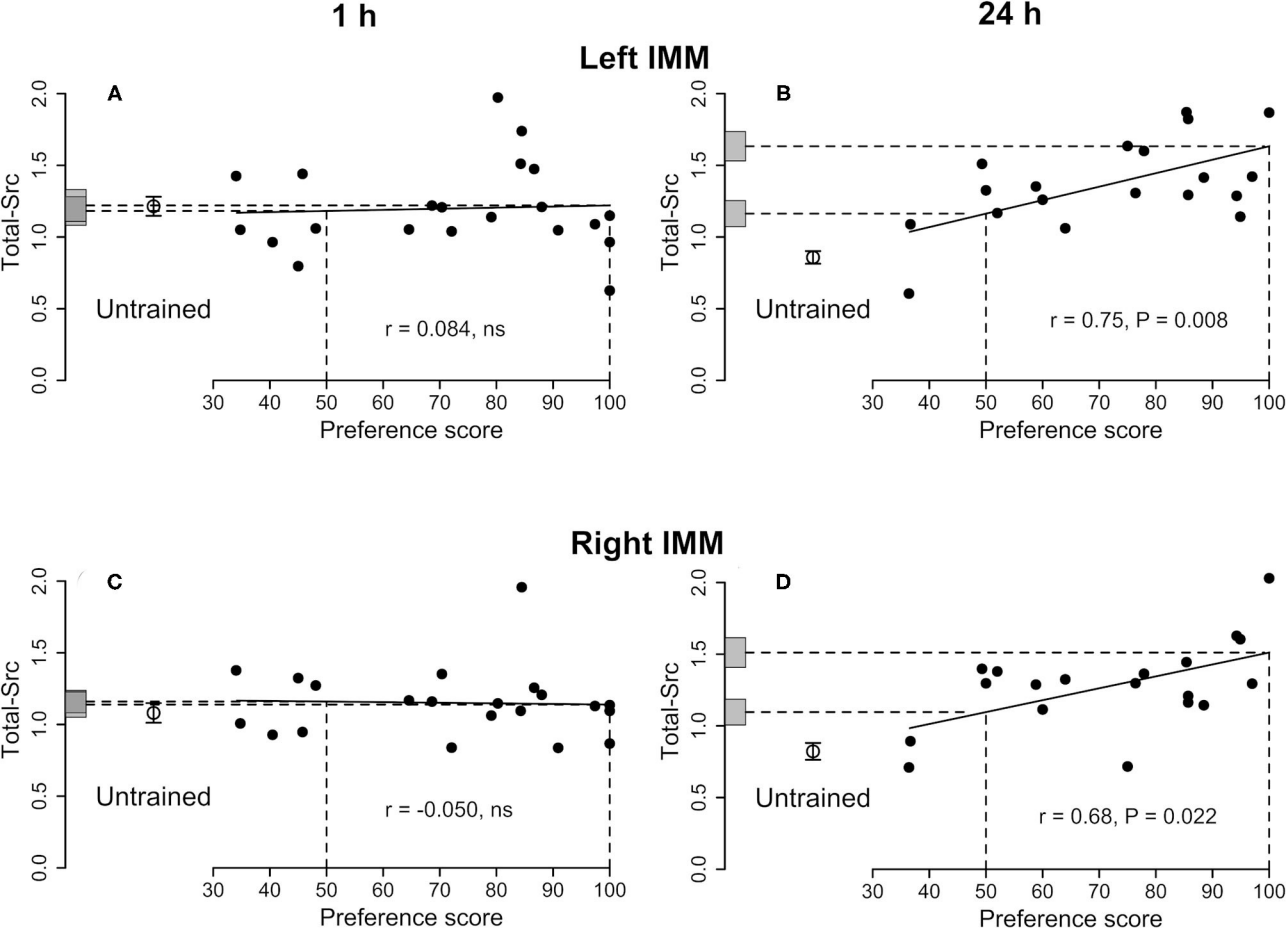
Left IMM and right IMM, 1 and 24 h after the end of training. Standardized relative amount of Total-Src plotted against preference score. Filled circles, trained chicks. Open circles and associated bars, mean level in untrained chicks ± SEM. Vertical dashed lines, preference scores 50 (no preference/no learning) and 100 (approach only to the training stimulus /strong learning); horizontal dashed lines, *y*-axis intercepts for preference scores 50 and 100; gray bars on *y*-axis, ± SE of intercept. **(A)** Left IMM and **(C)** right IMM 1 h after the end of training. Correlations are not significant. **(B)** Left IMM and **(D)** right IMM 24 h after the end of training. For both sides, the correlations are significant and the differences between the untrained mean and the intercept at preference score 100 are also significant. The intercepts at preference score 50 are also significantly higher than the mean of untrained chicks, indicating an effect of training on Total-Src in addition to a learning-related effect.

**Figure 2:**
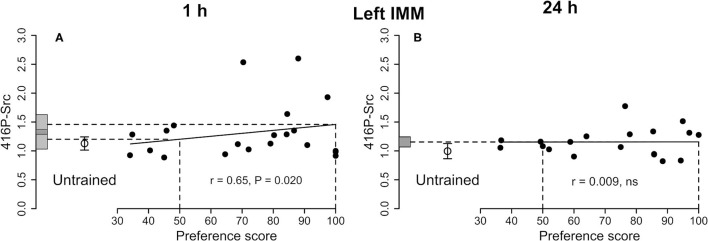
Left IMM. Standardized relative amount of 416P-Src plotted against preference score 1 h **(A)** and 24 h **(B)** after the end of training. Conventions otherwise as for Figure 1A, the correlation is significant; the mean value for untrained chicks is not significantly different from either intercept on the *y*-axis. **(B)** Correlation not significant.

**Figure 3:**
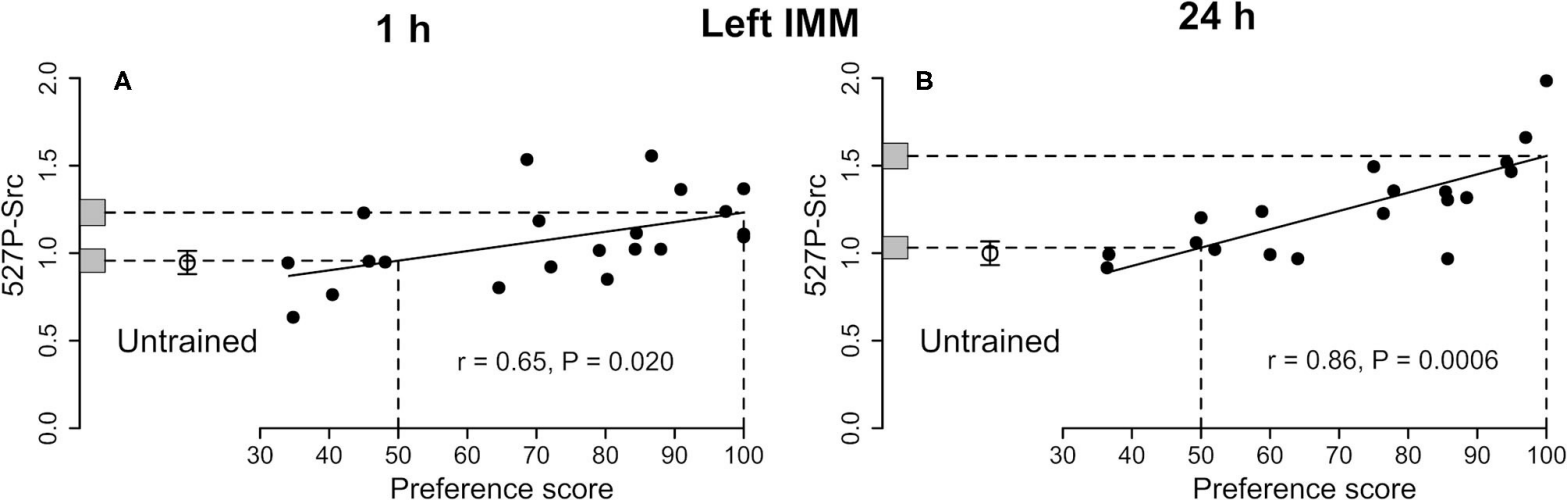
Left IMM. Standardized relative amount of 527P-Src plotted against preference score 1 h **(A)** and 24 h **(B)** after the end of training. Conventions otherwise as for Figure 1. For both times the correlations are significant. In each plot, the mean value for untrained chicks is significantly lower than the intercept at preference score 100 and not significantly different from the intercept at preference score 50.

**Figure 4:**
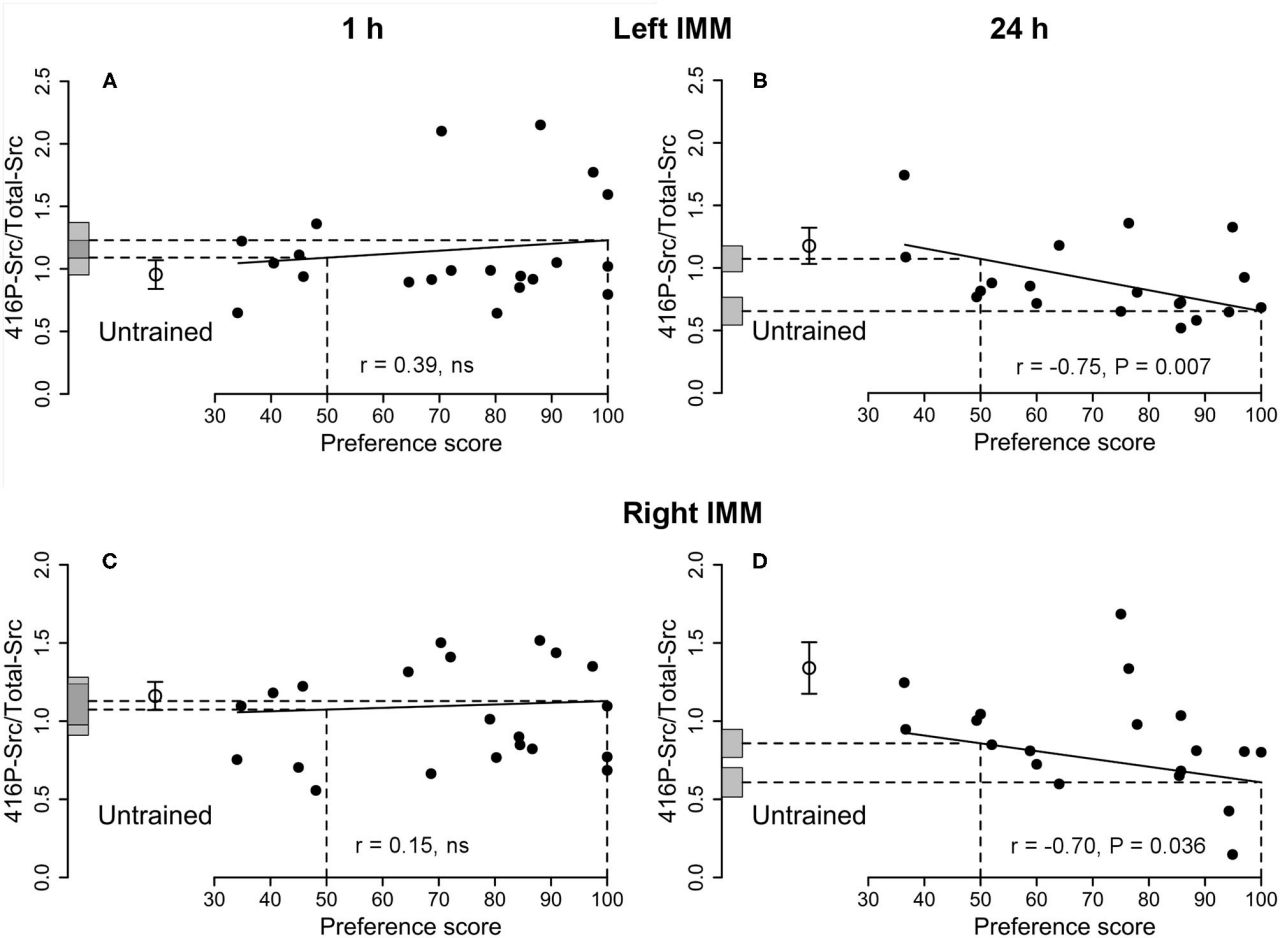
Left and right IMM, 1 and 24 h after the end of training. 416P-Src/Total-Src plotted against preference score. Conventions as for Figure 1. **(A,B)** Left IMM; **(C,D)** right IMM; **(A,C)** 1 h after the end of training, correlations not significant. **(B,D)** Twenty four hours after the end of training, both correlations significant. In both left and right IMM at 24 h **(B,D)**, the intercept at preference score 100 is significantly different from the mean of untrained chicks. In the right IMM at 24 h **(D)**, the intercept at preference score 50 is also significantly different from the mean of untrained chicks. Data in **(D)** are corrected for effects of approach during training and testing, which were statistically significant (cf. Supplementary Table S4).

A linear mixed-effects regression model was fitted to the relative amount of protein, with fixed term preference score and random terms chick nested within batch. The analysis was conducted using the lme function in the nlme package (Pinheiro et al., 2021) of R (R Development Core Team, 2016). The R script used for statistical analysis was essentially that published by Margvelani et al. (2018a). Data from the left and the right IMM and the left and the right PPN were analyzed separately owing to the functional disparity of the IMM and PPN, where the IMM is essential for imprinting and the PPN is not (Solomonia et al., 1997, 1998, 2000, 2005, 2011, 2013; Margvelani et al., 2018a). The two sides were analyzed separately because there is a functional hemispheric asymmetry in IMM with respect to imprinting, reviewed in Solomonia and McCabe (2015), including neurobiological consequences of learning; to date, when bilateral effects of learning in the IMM occur, they have been found predominantly on the left side (reviewed in Solomonia and McCabe, 2015). Results are summarized in Tables 1, 2 and Supplementary Tables S1–S6. Approach during training and approach during testing were added to the regression model as covariates in a further series of analyses. When preference score and one or both approach terms were significant in these analyses, or when the addition of one or both significant covariates caused the preference score term to lose significance, this is additionally reported in Results. For the figures showing an association between protein amount and preference score, variation attributable to differences between batches has been removed from the relative amount of protein by subtracting the estimated effect of batch (batch mean – overall mean) from each value, as occurred in the statistical analysis. This quantity is referred to as “the standardized relative amount of protein.” A regression line was fitted to the plot of the standardized relative amount of protein vs. preference score, between the lowest and highest preference score achieved (see Figures 1–**4**). The regression line was used to estimate protein levels corresponding to (i) preference score 50 (no learning) and (ii) the maximum preference score attained in the experiment (indicating strong learning; in the present experiments this preference score was 100).

**Table 1:** Standardized relative amount of protein.

**Brain region**	**Left IMM**					
**Protein**	**Total-Src**	**416P-Src**	**527P-Src**	**416P-Src/Total-Src**	**527P-Src/Total-Src**	**527P-Src/416P-Src**
**Untrained chicks**						
Mean	1.21	1.12	0.94	0.95	0.81	1.01
SEM	0.066	0.11	0.066	0.11	0.08	0.23
*DF*	9	9	9	9	9	9
**Trained chicks**						
Correlation protein amount vs. preference score	0.084	0.65 (0.84)	0.65	0.39	0.62	0.35
*DF*	10	10 (8)	10	10	10	0.1
*P*	0.79	0.02 (0.022)*	0.02*	0.2	0.031*	0.25
y-intercept at preference score 100	1.22	1.43 (1.46)	1.23	1.22	1.08	0.945
SE of y-intercept	0.11	0.16 (0.17)	0.07	0.14	0.1	0.1
**Comparison. y- intercept at preference score 100 vs. mean for untrained chicks**						
*t*	0.046	1.57 (1.61)	2.85	1.50	2.01	−0.26
*DF*	16.14	17.62 (14.26)	18.92	18.54	18.58	12.67
*P*	0.96	0.13 (0.13)	0.01*	0.15	0.058	0.79
y-intercept at preference score 50	1.18	1.24 (1.20)	0.095	1.08	0.85	0.84
SE of y-intercept	0.09	0.15 (0.17)	0.066	0.13	0.1	0.1
**Comparison. y-intercept at preference score 50 vs. mean for untrained chicks**						
*t*	−0.27	0.60 (0.36)	0.11	0.75	0.32	−0.67
*DF*	15.45	15.89 (12.65)	17.58	16.74	16.85	18.97
*P*	0.78	0.55 (0.73)	0.91	0.46	0.74	0.51
Residual regression variance/variance untrained	1.84	0.13 (0.08)	0.88	0.29	0.41	0.048
*P*	0.81	0.002 (0.0008)*	0.42	0.033*	0.096	0.00002*

*Summary of results for the left IMM 1 h after the end of training. For 416P-Src, approach during training and testing were significant; numbers in brackets give results when these approach terms were included in the regression model. Data for untrained chicks are in the upper part of the table and data from trained chicks below. y-Intercepts for preference scores 50 and 100 are given, together with results of comparisons of these intercepts with mean values for untrained chicks using t-tests. On the bottom line is given the probability (F-test) for a comparison of residual variance from the regression with the variance of untrained chicks. Asterisks indicate statistically significant results*.

**Table 2:** Standardized relative amount of protein.

**Brain region**	**Left IMM**					
**Protein**	**Total-Src**	**416P-Src**	**527P-Src**	**416P-Src/Total-Src**	**527P-Src/Total-Src**	**527P-Src/416P-Src**
**Untrained chicks**						
Mean	0.857	0.995	0.999	1.17	1.19	1.084
SEM	0.043	0.131	0.067	0.14	0.10	0.091
*DF*	8	8	8	8	8	8
**Trained chicks**						
Correlation protein amount vs. preference score	0.748	0.009	0.86	−0.75 (−0.83)	0.02	0.86
*DF*	9	9	9	9 (7)	9	9
*P*	0.008*	0.97	0.0006*	0.007 (0.005)*	0.94	0.0006*
y-intercept at preference score 100	1.63	1.156	1.55	0.65 (0.69)	0.95	1.399
SE of y-intercept	0.1	0.089	0.07	0.11 (0.13)	0.084	0.093
**Comparison. y- intercept at preference score 100 vs. mean for untrained chicks**						
*t*	6.94	1.01	5.59	−2.86 (−3.97)	−1.84	2.40
*DF*	12.15	14.37	16.99	15.4 (14.94)	15.96	16.97
*P*	0.00001*	0.32	0.00003*	0.011 (0.023)*	0.08	0.027*
y-intercept at preference score 50	1.16	1.15	1.03	1.07 (1.13)	0.94	0.90
SE of y-intercept	0.09	0.084	0.06	0.10 (0.12)	0.074	0.087
**Comparison. y- intercept at preference score 50 vs. mean for untrained chicks**						
*t*	3.02	1.01	0.34	−0.58 (−0.24)	−1.98	−1.42
*DF*	12.25	16.77	15.99	16.64 (13.52)	16.63	15.97
*P*	0.01**	0.32	0.73	0.56 (0.82)	0.06	0.17
Residual regression variance/variance untrained	3.22	0.10	0.74	0.211 (0.18)	0.42	0.336
*P*	0.94	0.001*	0.33	0.016 (0.017)*	0.11	0.06

*Summary of results for the left IMM 24 h after the end of training. Format and conventions as for Table 1. For 416P-Src/Total-Src, approach during training and testing were significant; numbers in brackets give results where these approach terms were included in the regression model*.

*Asterisks indicate statistically significant results*.

In interpreting the results, we first enquired whether protein level was significantly correlated with preference score, the measure of learning. This correlation was a necessary criterion for inferring learning-dependent change of protein level during training. However, a correlation can be interpreted in an alternative way, namely that protein level does not change during learning but reflects a predisposition to learn readily. In this second case, variation in protein level would contribute to the correlation/regression, thereby reducing the residual variance about the regression line (Margvelani et al., 2018a). A predisposition was therefore identified by (i) a significant correlation between protein level and preference score; (ii) no significant increase in the total variance of protein level in trained, relative to untrained chicks; (iii) a significant reduction of the residual variance of protein level in trained chicks relative to the variance in untrained chicks. If there were no significant reduction in residual variance, a learning-dependent association with preference score was inferred. See Discussion and Margvelani et al. (2018a) for further details.

Probabilities <0.05 were taken as significant and statistical tests are two-tailed, unless stated otherwise.

## Results

### Behavior

Samples were taken 1 h after the end of training from 21 trained chicks and their associated untrained controls, and samples were taken 24 h after training from 19 trained chicks and their untrained controls. Mean preference scores were 72.1 ± 4.9 SEM and 72.0 ± 4.7 SEM, respectively. For both time points the mean preference score was significantly higher than the “no preference” score of 50 (*t*-test, *P* < 0.0001 in both cases). Mean approach during training and testing was 64.2 ± 15.0 and 13.0 ± 2.3 m, respectively for the 1-h experiments, and 105.0 ± 25.0 and 26.1 ± 7.1 m, respectively for the 24-h experiments.

### Immunostaining

All three antibodies reacted with a protein band of 60 kDa molecular weight corresponding to total Src (Total-Src), 416P-Src, and 527P-Src (Supplementary Figure S1). Four standards, 15, 30, 45, and 60 μg of total protein corresponding to 0.5, 1.0, 1.5, and 2.0 relative amounts of protein, respectively, were applied to each gel. For these standards the optical densities of the immunostained bands (Total-Src, 416P-Src, and 527P-Src) were plotted against the amount of protein; in all standards, least-squares regression showed a good fit to a straight line (Supplementary Figure S1).

### One Hour After the End of Training

#### Left IMM

##### Total-Src

See Figure 1A and Table 1. No correlations were significant and thus no learning-related changes were detected.

##### 416P-Src

See Figure 2A and Table 1. The correlation with preference score as the only predictor variable was significant. When both training approach and testing approach were added to the model, the significant correlation persisted and both approach terms were significant [preference score *F*_(1,8)_ = 19.41, *P* = 0.0023; training approach *F*_(1,8)_ = 5.40, *P* = 0.049, testing approach *F*_(1,8)_ = 5.40, *P* = 0.044]. The difference between the intercept at the maximum preference score and untrained mean was not significant and the variance about the regression line was significantly lower than the variance of the untrained chicks. The total variance in trained chicks was not significantly different from untrained values. Taken together, these results are consistent with a predisposition, namely a correlation between 416P-Src level and capacity to learn, rather than a consequence of learning during training (see Discussion).

##### 527P-Src

See Figure 3A and Table 1. The correlation with preference score was significant. The difference between the intercept at the maximum preference score and untrained mean was also significant. The mean of the untrained chicks and the intercept at the preference score 50 were nearly identical. The variance about the regression plot was not significantly different from the variance of untrained chicks. Taken together, the results suggest that the correlation with preference score is attributable to learning that occurred during training (see Discussion).

##### 416P-Src/Total-Src

See Figure 4A and Table 1. The correlation was not significant and there was thus no evidence of a learning-related effect.

##### 527P-Src/Total-Src

See Table 1 and Supplementary Figure S2. The correlation between this ratio and preference score was significant. However, the intercept at maximum preference was not significantly different from the mean of untrained chicks and the data were not sufficient to support inference of an effect of learning.

##### 527P-Src/416-P-Src

See Table 1. The correlation between this ratio and preference score was not significant, providing no evidence for an association with learning.

#### Right IMM

See Supplementary Table S1 and, for Total-Src and 416P-Src/Total-Src, Figures 1C, 4C, respectively. No correlations were significant and there was thus no evidence of learning-related changes.

#### Left PPN

##### 527P-Src

See Supplementary Table S2. There was a marginally significant correlation between preference score and the standardized amount of Src protein phosphorylated at tyrosine-527. The difference between the intercept at the maximum preference score and untrained mean was not significant and there was thus insufficient evidence of a learning-related process.

No other correlations were significant (see Supplementary Table S2).

#### Right PPN

See Supplementary Table S3. There were no significant correlations with preference score and thus no evidence of learning-related changes.

#### Summary of Changes 1 H After Training

According to our criteria for inference of learning-dependent changes (see Discussion), changes 1 h after the end of training attributable to learning were detected only for 527P-Src in the left IMM. The results indicate that the significant correlation of 416P-Src with preference score in the left IMM can be accounted for by a predisposition to learn (see Discussion).

### Twenty Four Hours After the End of Training

#### Left IMM

##### Total-Src

See Figure 1B and Table 2. The total amount of Src increased significantly with preference score. The intercepts at both preference score 50 and maximum preference score were significantly higher than the mean value for untrained chicks. The residual variance from the regression with preference score was not significantly different from the variance of untrained chicks. Taken together, these results indicate that the correlation in the left IMM arose as a result of learning and that, in addition, training increased the amount of Total-Src when no learning had occurred (see Discussion).

##### 416P-Src

See Figure 2B and Table 2. The correlation with preference score was not significant, giving no evidence of a learning-related change.

##### 527P-Src

See Figure 3B and Table 2. The amount of Src protein phosphorylated at tyrosine-527 was significantly correlated with the preference score. The intercept at the maximum preference score significantly exceeded the mean level for untrained chicks. The intercept at preference score 50 and mean value of untrained chicks were not significantly different from each other. The residual variance from the regression plot and variance of untrained chicks were statistically homogeneous, indicating that, as with Total-Src, the increase in 527P-Src amount with preference score was a result of learning.

##### 416P-Src/Total-Src

See Figure 4B and Table 2. A significant negative correlation was found. The intercept of the maximum preference score was significantly lower than the mean for untrained chicks, which was not significantly different from the intercept value at preference score 50. Residual variance from the regression with preference score was significantly lower than the variance of untrained chicks, whereas the total variance in trained chicks was statistically homogeneous with the untrained chick values. These data support the hypothesis (see Discussion) that this correlation did not arise as a result of training but reflects a predisposition to learn existing in the absence of training.

##### 527P-Src/Total-Src

See Table 2. For this ratio, no correlation was significant, indicating that the learning-related increase in Total-Src was mainly or entirely attributable to the increase in amount of its 527P-Src form.

##### 527P-Src/416P-Src

See Table 2. The ratio of the two phosphorylated forms of Src increased significantly with preference score, predictably since 527P-Src increased and 416P-Src decreased as learning increased. The difference between the intercept at the maximum score was significantly higher than the mean of untrained chicks and the intercept at preference score 50 was not significantly different from the untrained mean. The residual variance from the regression plot and the variance of the untrained chicks were not significantly different from each other. It is concluded that, as in the case of Total-Src and 527P-Src, the ratio 527P-Src/416P-Src is a function of the strength of learning (see Discussion).

#### Right IMM

##### Total-Src

See Figure 1D and Supplementary Table S4. The amount of protein increased significantly with the preference score. Both intercepts, at preference score 50 and maximum preference score, were significantly greater than the mean of the untrained chicks. Statistical homogeneity of residual variance from the regression plot and variance of untrained chicks indicates that the increase in Total-Src amount with preference score is attributable to learning. The significant difference between the mean of untrained chicks and preference score 50 indicates that, in addition, training increased the amount of Total-Src even when learning did not occur (see Discussion).

##### 416P-Src/Total-Src

See Figure 4D and Supplementary Table S4. There was no significant correlation between the amount of protein and preference score when the preference score was the only predictor variable. However, there was a significant correlation when training approach and testing approach were included as covariates: all three terms were significant [preference score *F*_(1,7)_ = 6.67, *P* = 0.036; training approach *F*_(1,7)_ = 8.61, *P* = 0.022, testing approach *F*_(1,7)_ = 27.64, *P* = 0.0012]. Residual variance about the regression line was significantly lower than the variance of untrained chicks, indicating that 416P-Src/Total-Src was associated with a predisposition to learn readily.

There were no further significant correlations between the amount of protein and the preference score (see Supplementary Table S4).

#### Left PPN

See Supplementary Table S5. There were no significant correlations between the amount of protein and preference score and therefore no evidence of learning-related effects.

#### Right PPN

See Supplementary Table S6. There were no significant correlations between the amount of protein and preference score and therefore no evidence of learning-related effects.

#### Summary of Changes 24 H After Training

Twenty-four hours after the end of training a learning-dependent increase was found in the total amount of Src in both the left and right IMM. Furthermore, training in the absence of learning was associated with an increase in the amount of Total-Src. In the left IMM 527P-Src was increased in a learning-dependent way, whereas the ratio 527P-Src/Total-Src was not changed, indicating that the increase in the total amount of enzyme was attributable at least principally to changes in its inhibited form.

The results for 416P-Src/Total-Src in both left and right IMM (when the latter was corrected for approach during training and testing) indicate that these quantities reflected predispositions to learn.

## Discussion

We have elucidated the role of Src and its two phosphorylated forms in the memory of visual filial imprinting. Our analysis has discriminated between (i) neurobiological changes specifically related to memory and (ii) a predisposition manifest in neural changes attributable to a capacity to learn, rather than a result of learning itself.

To determine whether an effect of training is specifically related to learning, we have used the criteria explained in previous papers (McCabe, 2013; Solomonia and McCabe, 2015; Margvelani et al., 2018a). Briefly, a significant correlation is required between the protein level and the preference score (i.e., learning), such that the protein level corresponding to the maximum preference score (strong learning) is significantly different from the mean for untrained chicks. The protein level for trained chicks at preference score 50 (no learning) indicates whether incidental correlates of learning, such as locomotion and vocalization, have generated side-effects unrelated to learning. If the protein level at preference score is 50 for trained chicks, then no side-effects are evident. A difference between these two levels plus a correlation is evidence for both learning-related changes and side-effects unrelated to learning, which may then be distinguished from one another.

A further question is whether correlation with preference score is a result of learning or results from a predisposition to learn better, irrespective of training. To distinguish between these two possibilities, we have used an additional criterion, namely whether residual variance in the regression with preference score is at least as large as the variance in untrained chicks. If the correlation were a result of learning, one would expect the residual variance from the regression to be at least as large as the variance in untrained chicks, and learning would not be expected to lower this baseline variability. Conversely, if protein levels reflect a readiness to learn and are unaffected by training (a predisposition), one would expect the total variances of trained and untrained chicks to be similar, and in trained chicks some of the variance to be attributable to the correlation; this would lower the residual variance in trained chicks; see also Margvelani et al. (2018a).

### Properties and Pathways of Src

Phosphorylation of the tyrosine-527 residue of Src inactivates the enzyme by binding tyrosine-527 to the Src homology 2 domain, causing a conformational change that restricts substrate access to the kinase domain of Src (Xu et al., 1997; reviewed by Amata et al., 2014). Dephosphorylation of Tyr527 is followed by autophosphorylation at Tyr416, which leads to full activation of Src. Thus, the available data indicate that Src is not phosphorylated simultaneously on the 527 and 416 tyrosine residues and that changes in 416P-Src and 527P-Src therefore presumably reflect activated and inhibited forms, respectively, in different pools of Src. These pools could be in different cells or in different compartments of the same cell.

Phosphorylation of Src at Tyr527 is catalyzed by C-terminal Src kinase (Csk) (Okada, 2012), which attenuates NMDA-gated currents via the inhibition of Src (Socodato et al., 2017). Phosphorylation of Src at Tyr416 leads to activation of Src and phosphorylation of several synaptic proteins, including components of the NMDA receptor complex (Rajani et al., 2021). The two pools of Src thus represent different states of critical neurobiological pathways.

In addition to phosphorylation at Tyr-416 and Tyr-527, Src is a substrate for serine/threonine kinases, including protein kinase C (at Serine-12), protein kinase A (at Serine-17), and CDK1/cdc2 (at Threonine-34, Threonine-46, and Serine-72) (Roskoski, 2005). The physiological importance of these phosphorylation sites in the brain are incompletely understood.

### Learning-Dependent Changes in the Left IMM

#### One Hour After the End of Training

The above criteria for learning-dependence were satisfied for 527P-Src in the left IMM (Figure 3A). There was no significant correlation for Total-Src, and the ratio 527P-Src/Total-Src behaved similarly to 527P-Src alone, with the exception that the level of this ratio at maximum preference score was significantly greater than the mean for untrained chicks only at the *P* < 0.1 level (Table 1).

527P-Src is an inhibited form of the enzyme. Since there was no significant change in Total-Src, and since both 527P-Src and the ratio of 527P/Total-Src increased in learning-dependent ways in the left IMM, training evidently converted part of the pool of unphosphorylated Src to its inhibited form in proportion to the strength of learning. Learning and memory at 1 h are thus associated with a reduction of Src activity in the left IMM and may permit activity in pathways which are inhibited by active Src. A kinase strongly implicated in Tyr-527 phosphorylation, and which may have been responsible for generation of 527P-Src, is Csk (Okada, 2012; Amata et al., 2014).

#### Twenty Four Hours After the End of Training

Total-Src in the left IMM satisfied the criteria for learning-dependence whereas the level corresponding to preference score 50 was significantly greater than the untrained value (Figure 1B). Therefore, the amount of Total-Src in the left IMM was evidently increased by learning-dependent processes and by separate processes unconnected with learning. The change in Total-Src level with increasing preference score was accompanied by a learning-dependent increase in 527P-Src (Figure 3B). The similar relation of each of these two measures to preference score resulted in their ratio not showing a significant correlation (Table 2).

The level of 416P-Src did not change significantly with preference score (Figure 2B), with the result that the ratio 527P-Src/416P-Src increased in the left IMM in a learning-dependent way (Table 1).

As at 1 h, the increase in 527P-Src with increasing preference score indicates more inhibition of Src activity as memory becomes stronger. At this later time, stronger memory is also associated with a greater amount of Total-Src, that is, Src available for activation and modulation via its phosphorylation sites.

### Results in the Left IMM Implying a Predisposition

#### One Hour After the End of Training

A significant positive correlation was found for 416P-Src (Figure 2A), but the residual variance from the regression was significantly lower than the variance in untrained chicks (Table 1), implicating 416P-Src at this time after training in a predisposition reflects the readiness of chicks to learn.

#### Twenty Four Hours After the End of Training

Although there was no significant correlation for 416P-Src alone, the ratio 416P-Src/Total-Src was negatively correlated with preference score in both the left and the right IMM (Figures 4B,D). The residual variance from the regression was significantly lower than the variance in untrained chicks (Table 2 and Supplementary Table S4), implicating 416P-Src in a predisposition at 24 h as well as at 1 h. However, the pattern of variation with preference score was different at the two times, suggesting a time-dependent, possibly age-related, change in the role of Src in the predisposition. In addition, there was, in the right IMM at 24 h and after correction for approach, a significant difference between the intercept at preference score 50 and the mean of untrained chicks (Figure 4D and Supplementary Table S4). There was thus an additional effect of training, delayed and unrelated to learning, in the right IMM.

There is a precedent for a predisposition of the type discussed above, where the expression of the micro-RNA gga-miR-130b-3p in the left IMM was found to be inversely correlated with preference score, and its residual variance about the regression line was significantly lower than the variance of untrained control chicks (Margvelani et al., 2018a). The small, non-coding sequence of this micro-RNA regulates post-transcriptional expression of a number of proteins, including cytoplasmic polyadenylation element binding protein 3 (CPEB-3). Considering our previous findings together with the results of the present study, it appears likely that an array of biochemical conditions accompanies a predisposition to learn readily. Other predispositions, which can act in concert with imprinting, have been described (reviewed by Rosa-Salva et al., 2021) but the extent to which neurobiological mechanisms are shared by these processes is unknown.

It is of interest that learning-dependent processes and processes associated with a predisposition are associated with different patterns of Src phosphorylation. It is also of interest that processes reflecting both learning and a predisposition to learn occur in the left IMM, where there may be opportunities for interactions between the two types of process. Relevant to this result is the observation (Mayer et al., 2016), of a change in expression of the neuronal activity marker c-fos protein in the IMM in association with another predisposition—a preference for facial features of an adult bird (Johnson and Horn, 1988; Rosa-Salva et al., 2010). Interaction between predispositions and memory in the IMM might increase the adaptive advantage of behavior learned via imprinting, even though the IMM is not necessary for the predisposition to prefer faces (Horn and McCabe, 1984; Johnson and Horn, 1986).

Src activation increases NMDA currents in hippocampal cultured neurons (Yu et al., 1997) and Src inhibition reduces the surface expression of NR2B receptors and synaptic plasticity in the amygdala (Sinai et al., 2010). NMDA receptors and glutamate release in the left IMM are critically involved in the learning and memory of imprinting (McCabe and Horn, 1988, Meredith et al., 2004), NMDA receptor-dependent synaptic plasticity has been demonstrated in the IMM (Bradley et al., 1993; Matsushima and Aoki, 1995), and it is possible that Src contributes to these processes.

Overexpression of constitutively active Src suppresses Ca^2+^-induced release of neurotransmitter (Ohnishi et al., 2011). Knockout of Src homolog domain-containing phosphatase 2 (Shp2) in hippocampal pyramidal neurons leads to the activation of Src with the disruption of excitatory synaptic transmission and impairment of remote fear memory in mice (Yan et al., 2017). Knockdown of cellular Src in primary cortical neurons protects cells against glutamate-induced loss of viability (Khanna et al., 2007). It appears, therefore, that excessive Src activity can be maladaptive. Given the importance of Src in memory demonstrated by the present results, it is possible that for the proper functioning of Src in synaptic plasticity it is necessary for a balance of activated and inhibited forms of the enzyme to be maintained, disturbance of which could lead to disruption of neuronal homeostasis.

### Regional Specificity

Learning-related changes in Src were found in the IMM and not in the PPN control forebrain region, reflecting a regional specificity found in all previous studies of imprinting in which measurements have been made on these two regions; see e.g., Solomonia and McCabe (2015).

### Hemispheric Asymmetry

The left and right IMM have different roles in imprinting, which are reflected in functional hemispheric asymmetries following training (reviewed by Horn, 1985; McCabe, 2013, 2019). Evidence from ablation experiments indicates that both the left and the right IMM have a storage function but that, in addition, the right IMM is necessary for subsequent storage without the IMM, in an, as yet, unidentified region termed S' (Cipolla-Neto et al., 1982); as shown in Solomonia et al. (2000) and Tiunova et al. (2019) for possible candidate regions for S'. Learning-related biochemical changes are usually more strongly expressed in the left IMM than in the right IMM, especially 24 h after imprinting (Solomonia and McCabe, 2015; see also Margvelani et al., 2018a; Chitadze et al., 2020). The present study provides additional evidence of hemispheric asymmetry. In the left IMM, significant learning-dependent changes in 527P-Src were observed at both 1 h and 24 h (Figure 3 and Tables 1, 2), but not in the right IMM (Supplementary Tables S1, S4).

A learning-dependent change in Total-Src was detected in both left and right IMM 24 h after the end of training, providing further evidence for the involvement of the right IMM in memory. Learning thus leads to a delayed increase in the amount of Total-Src in both hemispheres, evidently contributing to their different roles via differential phosphorylation.

### Conclusion

The present results demonstrate that Src protein contributes to memory formation and a predisposition to learn in ways that are time-dependent and regionally specific. The total amount of Src and its two phosphorylated conditions, one linked to activation and another to inhibition of the enzyme, have been shown to be involved. As far as we know, this is the first comprehensive study of Src and its phosphorylated forms in relation to learning and memory.

## Data Availability Statement

The original contributions presented in the study are included in the article/Supplementary Material. Further inquiries can be directed to the corresponding authors.

## Ethics Statement

This animal study was reviewed and approved by the I. Beritashvili Centre of Experimental Biomedicine.

## Author Contributions

MM: major contribution to experimentation, experimental design, and data analysis. LC: experimentation, experimental design, and data analysis. VL and BM: data analysis and writing paper. RS: experimentation, experimental design, data analysis, and writing paper. All authors contributed to the article and approved the submitted version.

## Funding

This work was supported by the Sh. Rustaveli National Science Foundation, Georgia (Grant YS-18-692), European Union's Horizon 2020 research and innovation programme (project CHARM-Vis, project ID 867429), and Robinson College, University of Cambridge.

## Conflict of Interest

The authors declare that the research was conducted in the absence of any commercial or financial relationships that could be construed as a potential conflict of interest.

## Publisher's Note

All claims expressed in this article are solely those of the authors and do not necessarily represent those of their affiliated organizations, or those of the publisher, the editors and the reviewers. Any product that may be evaluated in this article, or claim that may be made by its manufacturer, is not guaranteed or endorsed by the publisher.

## Abbreviations

DF: degrees of freedom
SE: standard error
SEM: standard error of the mean
t: Student's t.
