# Supplementary material for: Src and Memory: A Study of Filial Imprinting and Predispositions in the Domestic Chick
Source: Front Physiol. 2021 Sep 20;12:736999. doi: 10.3389/fphys.2021.736999 (PMC8488273; doi:10.3389/fphys.2021.736999)
Supplement: Supplementary file 1 [file Table_1.docx]

Supplementary Table S1. Standardised relative amount of protein. Summary of results for the right IMM 1 h after the end of training. Data for untrained chicks are in the upper part of the table and data from trained chicks below. y-intercepts for preference scores 50 and 100 are given, together with results of comparisons of these intercepts with mean values for untrained chicks using *t*-tests. On the bottom line is given the probability (*F*-test) for a comparison of residual variance from the regression with the variance of untrained chicks. Asterisks indicate statistically significant results.

| Brain Region | Right IMM | | | | | |
| --- | --- | --- | --- | --- | --- | --- |
| Protein | **Total-Src** | **416P-Src** | **527P-Src** | **416P-Src/Total-Src** | **527P-Src/Total-Src** | **527P-Src/416P-Src** |
| Untrained chicks | | | | | | |
| Mean | 1.08 | 1.21 | 0.88 | 1.16 | 0.86 | 0.75 |
| s.e.m | 0.068 | 0.077 | 0.081 | 0.08 | 0.12 | 0.08 |
| Df | 9 | 9 | 9 | 9 | 9 | 9 |
| Trained chicks | | | | | | |
| Correlation protein amount vs preference score | -0.05 | 0.18 | 0.41 | 0.15 | 0.47 | 0.24 |
| Df | 10 | 10 | 10 | 10 | 10 | 10 |
| P | 0.87 | 0.55 | 0.18 | 0.62 | 0.11 | 0.44 |
| y-intercept at preference score 100 | 1.13 | 1.20 | 1.09 | 1.07 | 1.02 | 0.98 |
| SE y-intercept | 0.088 | 0.126 | 0.1 | 0.11 | 0.11 | 0.13 |
| Comparison. y- intercept at preference score 100 vs mean for untrained chicks | | | | | | |
| T | 0.519 | -0.08 | 1.58 | -0.61 | 0.96 | 1.45 |
| Df | 18.27 | 16.34 | 17.9 | 18.54 | 18.73 | 16.09 |
| P | 0.60 | 0.93 | 0.13 | 0.54 | 0.34 | 0.16 |
| y- intercept at preference score 50 | 1.159 | 1.12 | 0.087 | 0.99 | 0.75 | 0.83 |
| SE of Y-intercept | 0.077 | 0.117 | 0.09 | 0.09 | 0.09 | 0.12 |
| Comparison. y- intercept at preference score 50 vs mean for untrained chicks | | | | | | |
| T | 0.76 | -0.69 | -0.05 | -1.25 | -0.73 | 0.57 |
| Df | 17.02 | 15.39 | 16.78 | 17.24 | 18.26 | 15.50 |
| P | 0.45 | 0.49 | 0.95 | 0.22 | 0.47 | 0.57 |
| Residual regression variance/variance untrained | 1.34 | 0.62 | 1.48 | 1.22 | 0.70 | 2.02 |
| P | 0.66 | 0.34 | 0.71 | 0.61 | 0.29 | 0.86 |
