# Supplementary material for: Src and Memory: A Study of Filial Imprinting and Predispositions in the Domestic Chick
Source: Front Physiol. 2021 Sep 20;12:736999. doi: 10.3389/fphys.2021.736999 (PMC8488273; doi:10.3389/fphys.2021.736999)
Supplement: Supplementary file 2 [file Table_2.docx]

Supplementary Table S2. Standardised relative amount of protein. Summary of results for the Left PPN 1 h after the end of training. Data for untrained chicks are in the upper part of the table and data from trained chicks below. y-intercepts for preference scores 50 and 100 are given, together with results of comparisons of these intercepts with mean values for untrained chicks using *t*-tests. On the bottom line is given the probability (*F*-test) for a comparison of residual variance from the regression with the variance of untrained chicks. Asterisks indicate statistically significant results.

| Brain Region | Left PPN | | | | | |
| --- | --- | --- | --- | --- | --- | --- |
| Protein | **Total-Src** | **416P-Src** | **527P-Src** | **416P-Src/Total-Src** | **527P-Src/Total-Src** | **527P-Src/416P-Src** |
| Untrained chicks | | | | | | |
| Mean | 1.16 | 0.99 | 0.84 | 0.87 | 0.82 | 1.25 |
| s.e.m. | 0.12 | 0.11 | 0.08 | 0.1 | 0.14 | 0.35 |
| Df | 9 | 9 | 9 | 9 | 9 | 9 |
| Trained chicks | | | | | | |
| Correlation protein amount vs preference score | 0.08 | -0.27 | 0.58 | -0.3 | 0.36 | 0.67 |
| Df | 10 | 9 | 10 | 9 | 10 | 9 |
| P | 0.8 | 0.40 | 0.045* | 0.36 | 0.24 | 0.024* |
| y-intercept at preference score 100 | 1.18 | 1.05 | 1.02 | 0.9 | 0.88 | 1.54 |
| SE y-intercept | 0.06 | 0.13 | 0.07 | 0.13 | 0.09 | 0.13 |
| Comparison. y-intercept at preference score 100 vs mean for untrained chicks | | | | | | |
| T | 0.11 | 0.32 | 1.62 | 0.18 | 0.35 | -0.29 |
| Df | 14.4 | 17.36 | 18.55 | 16.68 | 15.8 | 11.68 |
| P | 0.91 | 0.74 | 0.12 | 0.85 | 0.72 | 0.78 |
| y- intercept at preference score 50 | 1.15 | 1.22 | 0.78 | 1.09 | 0.72 | 0.64 |
| SE of y-intercept | 0.06 | 0.12 | 0.06 | 0.12 | 0.08 | 0.12 |
| Comparison. y- intercept at preference score 50 vs mean for untrained chicks | | | | | | |
| T | -0.06 | 1.36 | -0.54 | 1.36 | -0.63 | -1.73 |
| Df | 18.95 | 15.64 | 18.35 | 15.15 | 18.84 | 17.93 |
| P | 0.94 | 0.19 | 0.59 | 0.19 | 0.53 | 0.10 |
| Residual regression variance/variance untrained | 0.26 | 1.18 | 0.64 | 1.42 | 0.35 | 0.14 |
| P | 0.02* | 0.59 | 0.24 | 0.69 | 0.058 | 0.002* |
