# Supplementary material for: Src and Memory: A Study of Filial Imprinting and Predispositions in the Domestic Chick
Source: Front Physiol. 2021 Sep 20;12:736999. doi: 10.3389/fphys.2021.736999 (PMC8488273; doi:10.3389/fphys.2021.736999)
Supplement: Supplementary file 3 [file Table_3.docx]

Supplementary Table S3. Standardised relative amount of protein. Summary of results for the Right PPN 1 h after the end of training. Data for untrained chicks are in the upper part of the table and data from trained chicks below. y-intercepts for preference scores 50 and 100 are given, together with results of comparisons of these intercepts with mean values for untrained chicks using *t*-tests. On the bottom line is given the probability (*F*-test) for a comparison of residual variance from the regression with the variance of untrained chicks. Asterisks indicate statistically significant results.

| Brain Region | Right PPN | | | | | |
| --- | --- | --- | --- | --- | --- | --- |
| Protein | **Total-Src** | **416P-Src** | **527P-Src** | **416P-Src/Total-Src** | **527P-Src/Total-Src** | **527P-Src/416P-Src** |
| Untrained chicks | | | | | | |
| Mean | 1.11 | 1.02 | 0.86 | 0.9 | 0.78 | 1.00 |
| s.e.m | 0.04 | 0.08 | 0.03 | 0.08 | 0.04 | 0.2 |
| Df | 8 | 8 | 8 | 8 | 8 | 8 |
| Trained chicks | | | | | | |
| Correlation protein amount vs preference score | -0.022 | -0.12 | 0.5 | -0.05 | 0.34 | 0.47 |
| Df | 10 | 10 | 10 | 10 | 10 | 10 |
| P | 0.94 | 0.69 | 0.9 | 0.85 | 0.27 | 0.11 |
| y-intercept at preference score 100 | 1.16 | 1.06 | 1.02 | 0.95 | 0.88 | 1.00 |
| SE y-intercept | 0.08 | 0.12 | 0.1 | 0.12 | 0.1 | 0.11 |
| Comparison. y- intercept at preference score 100 vs mean for untrained chicks | | | | | | |
| T | 0.48 | 0.23 | 1.49 | 0.29 | 0.89 | -0.01 |
| Df | 15.51 | 16.94 | 11.7 | 16.88 | 13.45 | 13.04 |
| P | 0.63 | 0.81 | 0.16 | 0.76 | 0.38 | 0.98 |
| y- intercept at preference score 50 | 1.17 | 1.13 | 0.77 | 0.98 | 0.71 | 0.72 |
| SE of Y-intercept | 0.07 | 0.1 | 0.09 | 0.1 | 0.09 | 0.1 |
| Comparison. y- intercept at preference score 50 vs mean for untrained chicks | | | | | | |
| T | 0.63 | 0.77 | -0.86 | 0.56 | -0.65 | -1.25 |
| Df | 15.14 | 16.12 | 11.86 | 16.08 | 13.59 | 17.99 |
| P | 0.53 | 0.44 | 0.4 | 0.58 | 0.52 | 0.22 |
| Residual regression variance/variance untrained | 2.74 | 1.89 | 8.01 | 1.92 | 4.85 | 0.29 |
| P | 0.91 | 0.81 | 0.99 | 0.81 | 0.98 | 0.038* |
