# Supplementary material for: Src and Memory: A Study of Filial Imprinting and Predispositions in the Domestic Chick
Source: Front Physiol. 2021 Sep 20;12:736999. doi: 10.3389/fphys.2021.736999 (PMC8488273; doi:10.3389/fphys.2021.736999)
Supplement: Supplementary file 4 [file Table_4.docx]

Supplementary Table S4. Standardised relative amount of protein. Summary of results for the Right IMM 24 h after the end of training. Data for untrained chicks are in the upper part of the table and data from trained chicks below. y-intercepts for preference scores 50 and 100 are given, together with results of comparisons of these intercepts with mean values for untrained chicks using *t*-tests. On the bottom line is given the probability (*F*-test) for a comparison of residual variance from the regression with the variance of untrained chicks. Asterisks indicate statistically significant results. Numbers in brackets: data after correction for significant effects of approach during training and testing.

| Brain Region | Right IMM | | | | | |
| --- | --- | --- | --- | --- | --- | --- |
| Protein | **Total-Src** | **416P-Src** | **527P-Src** | **416P-Src/Total-Src** | **527P-Src/Total-Src** | **527P-Src/416P-Src** |
| Untrained chicks | | | | | | |
| Mean | 0.822 | 1.04 | 1.039 | 1.33 (1.34) | 1.34 | 1.03 |
| s.e.m | 0.058 | 0.097 | 0.07 | 0.16 (0.16) | 0.16 | 0.09 |
| Df | 8 | 8 | 8 | 8 | 8 | 8 |
| Trained chicks | | | | | | |
| Correlation protein amount vs preference score | 0.675 | -0.09 | 0.47 | -0.488  (-0.70) | -0.47 | 0.39 |
| Df | 9 | 9 | 9 | 9 (7) | 9 | 9 |
| P | 0.022* | 0.78 | 0.14 | 0.12 (0.036*) | 0.13 | 0.23 |
| y-intercept at preference score 100 | 1.51 | 1.02 | 1.23 | 0.699 (0.61) | 0.81 | 1.63 |
| SE y-intercept | 0.10 | 0.14 | 0.06 | 0.126 (0.09) | 0.11 | 0.32 |
| Comparison. y- intercept at preference score 100 vs mean for untrained chicks | | | | | | |
| T | 5.77 | -0.14 | 1.99 | -3.08 (-3.9) | -2.67 | 1.79 |
| Df | 14.05 | 15.53 | 16.35 | 15.42 (12.6) | 14.68 | 10.44 |
| P | 0.00004* | 0.88 | 0.062 | 0.007* (0.002*) | 0.017* | 0.10 |
| y- intercept at preference score 50 | 1.09 | 1.08 | 1.09 | 1.008 (0.86) | 1.6 | 1.03 |
| SE of Y-intercept | 0.089 | 0.12 | 0.062 | 0.10 (0.089) | 0.10 | 0.27 |
| Comparison. y- intercept at preference score 50 vs mean for untrained chicks | | | | | | |
| T | 2.55 | 0.22 | 0.59 | -1.67 (-2.6) | -0.143 | 0.018 |
| Df | 13.80 | 14.78 | 16.52 | 16.75 (14.4) | 16.82 | 10.73 |
| P | 0.02 | 0.82 | 0.55 | 0.11 (0.02*) | 0.16 | 0.98 |
| Residual regression variance/variance untrained | 2.23 | 1.53 | 0.021 | 0.41 (0.10) | 0.30 | 8.81 |
| P | 0.86 | 0.72 | 0.12 | 0.1 (0.004*) | 0.049* | 0.99 |
