# Supplementary material for: Src and Memory: A Study of Filial Imprinting and Predispositions in the Domestic Chick
Source: Front Physiol. 2021 Sep 20;12:736999. doi: 10.3389/fphys.2021.736999 (PMC8488273; doi:10.3389/fphys.2021.736999)
Supplement: Supplementary file 5 [file Table_5.docx]

Supplementary Table S5. Standardised relative amount of protein. Summary of results for the left PPN 24 h after the end of training. Data for untrained chicks are in the upper part of the table and data from trained chicks below. y-intercepts for preference scores 50 and 100 are given, together with results of comparisons of these intercepts with mean values for untrained chicks using *t*-tests. On the bottom line is given the probability (*F*-test) for a comparison of residual variance from the regression with the variance of untrained chicks. Asterisks indicate statistically significant results.

| Brain Region | Left PPN | | | | | |
| --- | --- | --- | --- | --- | --- | --- |
| Protein | **Total-Src** | **416P-Src** | **527P-Src** | **416P-Src/Total-Src** | **527P-Src/Total-Src** | **527P-Src/416P-Src** |
| Untrained chicks | | | | | | |
| Mean | 0.889 | 1.045 | 1.04 | 1.22 | 1.15 | 1.23 |
| s.e.m | 0.057 | 0.14 | 0.075 | 0.20 | 0.09 | 0.25 |
| Df | 7 | 8 | 8 | 7 | 7 | 8 |
| Trained chicks | | | | | | |
| Correlation protein amount vs preference score | 0.44 | -0.40 | 0.2 | -0.47 | -0.13 | 0.22 |
| Df | 9 | 9 | 9 | 9 | 9 | 9 |
| P | 0.16 | 0.21 | 0.54 | 0.13 | 0.7 | 0.51 |
| y-intercept at preference score 100 | 1.49 | 1.10 | 1.16 | 0.76 | 0.81 | 1.08 |
| SE y-intercept | 0.099 | 0.088 | 0.07 | 0.09 | 0.09 | 0.11 |
| Comparison. y- intercept at preference score 100 vs mean for untrained chicks | | | | | | |
| T | 5.3 | 0.35 | 1.05 | -1.99 | -2.48 | -0.14 |
| Df | 14.06 | 13.48 | 16.99 | 9.88 | 15.84 | 11.26 |
| P | 0.0001** | 0.73 | 0.3 | 0.07 | 0.02 | 0.61 |
| y- intercept at preference score 50 | 1.28 | 1.18 | 1.09 | 0.930 | 0.87 | 0.97 |
| SE of Y-intercept | 0.085 | 0.086 | 0.68 | 0.08 | 0.08 | 0.1 |
| Comparison. y- intercept at preference score 50 vs mean for untrained chicks | | | | | | |
| T | 3.79 | 0.80 | 0.42 | -1.30 | -2.22 | -0.93 |
| Df | 13.77 | 16.85 | 16.11 | 15.97 | 15.60 | 16.99 |
| P | 0.002* | 0.43 | 0.67 | 0.21 | 0.041* | 0.36 |
| Residual regression variance/variance untrained | 2.36 | 0.046 | 0.79 | 0.077 | 0.85 | 0.13 |
| P | 0.86 | 0.00005** | 0.36 | 0.0004** | 0.4 | 0.003*** |
