# Supplementary material for: Src and Memory: A Study of Filial Imprinting and Predispositions in the Domestic Chick
Source: Front Physiol. 2021 Sep 20;12:736999. doi: 10.3389/fphys.2021.736999 (PMC8488273; doi:10.3389/fphys.2021.736999)
Supplement: Supplementary file 6 [file Table_6.docx]

Supplementary Table S6. Standardised relative amount of protein. Summary of results for the Right PPN 24h after the end of training. Data for untrained chicks are in the upper part of the table and data from trained chicks below. y-intercepts for preference scores 50 and 100 are given, together with results of comparisons of these intercepts with mean values for untrained chicks using *t*-tests. On the bottom line is given the probability (*F*-test) for a comparison of residual variance from the regression with the variance of untrained chicks. Asterisks indicate statistically significant results.

| Brain Region | Right PPN | | | | | |
| --- | --- | --- | --- | --- | --- | --- |
| Protein | **Total-Src** | **416P-Src** | **527P-Src** | **416P-Src/Total-Src** | **527P-Src/Total-Src** | **527P-Src/416P-Src** |
| Untrained chicks | | | | | | |
| Mean | 0.94 | 0.94 | 1.06 | 1.103 | 1.19 | 1.51 |
| s.e.m | 0.06 | 0.16 | 0.082 | 0.2 | 0.11 | 0.39 |
| Df | 8 | 7 | 7 | 7 | 7 | 7 |
| Trained chicks | | | | | | |
| Correlation protein amount vs preference score | 0.46 | -0.003 | 0.21 | -0.24 | -0.06 | 0.34 |
| Df | 9 | 9 | 9 | 9 | 9 | 9 |
| P | 0.15 | 0.99 | 0.52 | 0.46 | 0.84 | 0.29 |
| y-intercept at preference score 100 | 1.75 | 0.978 | 1.21 | 0.60 | 0.79 | 1.78 |
| SE y-intercept | 0.18 | 0.14 | 0.13 | 0.12 | 0.12 | 0.4 |
| Comparison. y- intercept at preference score 100 vs mean for untrained chicks | | | | | | |
| T | 4.21 | 0.13 | 0.89 | -2.05 | -2.43 | 0.48 |
| Df | 11.2 | 14.82 | 14.4 | 11.9 | 15.99 | 15.86 |
| P | 0.001* | 0.89 | 0.38 | 0.06 | 0.02* | 0.63 |
| y- intercept at preference score 50 | 1.34 | 0.98 | 1.08 | 0.74 | 0.82 | 1.13 |
| SE of Y-intercept | 0.15 | 0.12 | 0.11 | 0.10 | 0.10 | 0.34 |
| Comparison. y- intercept at preference score 50 vs mean for untrained chicks | | | | | | |
| T | 2.37 | 0.15 | 0.09 | -1.52 | -.239 | -0.73 |
| Df | 11.5 | 15.87 | 14.00 | 15.99 | 15.43 | 15.59 |
| P | 0.035* | 0.87 | 0.92 | 0.14 | 0.029 | 0.47 |
| Residual regression variance/variance untrained | 5.62 | 0.58 | 2.14 | 0.3 | 0.98 | 0.85 |
| P | 0.98 | 0.22 | 0.83 | 0.49 | 0.48 | 0.4 |
