# Supplementary material for: Src and Memory: A Study of Filial Imprinting and Predispositions in the Domestic Chick
Source: Front Physiol. 2021 Sep 20;12:736999. doi: 10.3389/fphys.2021.736999 (PMC8488273; doi:10.3389/fphys.2021.736999)
Supplement: Supplementary file 7 [file Data_Sheet_1.docx]

Supplementary Figure S1. Sample films and calibration plots for A, Total-Src; B, 416P-Src; C, 527P-Src. Top panels, calibration plots (lines fitted by linear least-squares regression). Bottom panels, internal standards sample film containing, respectively, 15, 30, 45, and 60 𝜇g protein, corresponding to 0.5, 1.0, 1.5 and 2.0 relative amounts of protein. For calibrations R-squared value was more than 97%.

Supplementary Figure S2. Left IMM, 24 h after the end of training. Standardized relative amount of 527P-Src/Total-Src plotted against preference score. Conventions as for Figure 1 in the main text. The correlation is significant. The intercept at maximum preference is not significantly different from the mean of untrained chicks.
