# Supplementary figures and images for: Src and Memory: A Study of Filial Imprinting and Predispositions in the Domestic Chick
Source: Front Physiol. 2021 Sep 20;12:736999. doi: 10.3389/fphys.2021.736999 (PMC8488273; doi:10.3389/fphys.2021.736999)

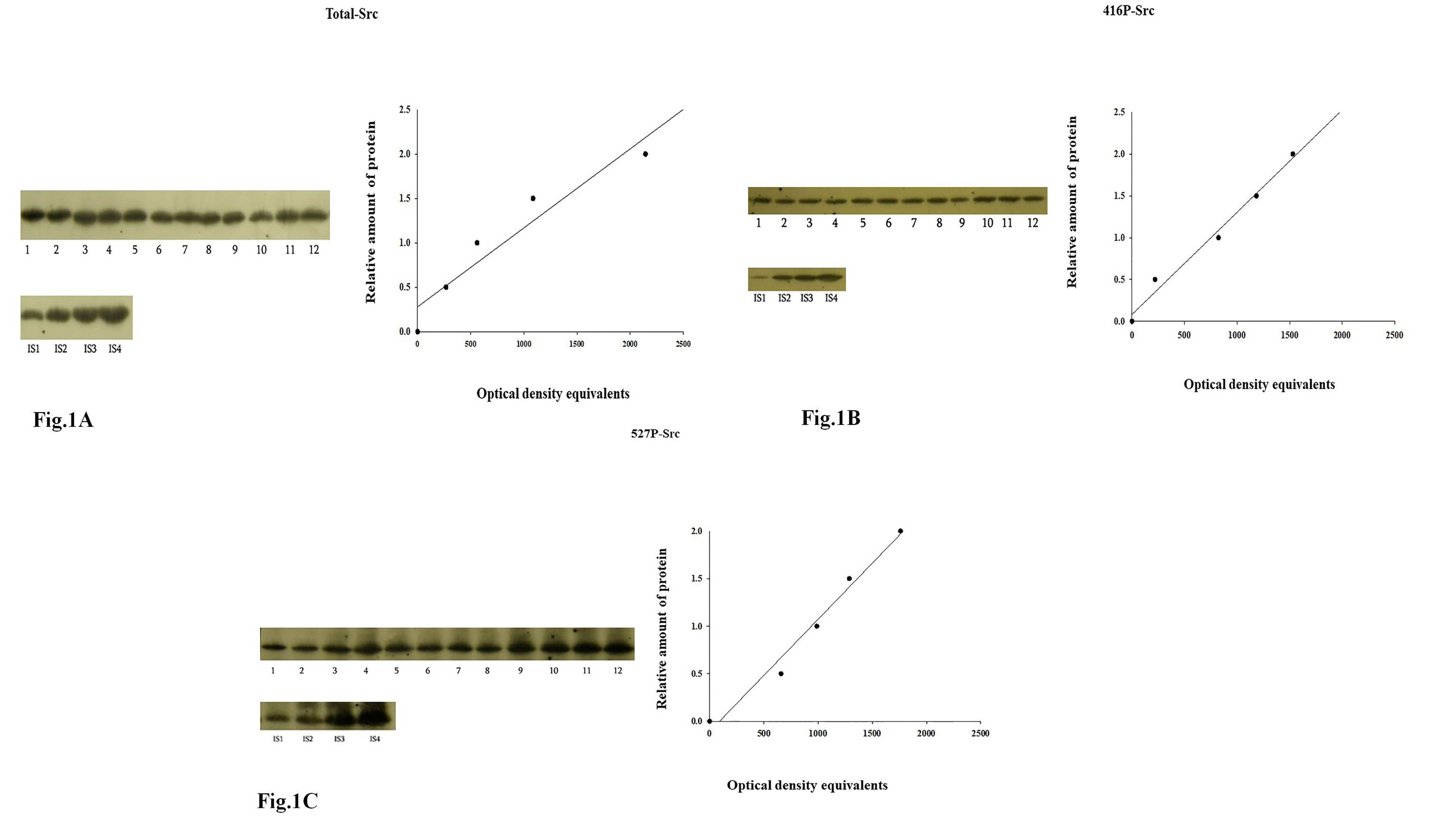

Supplement: Supplementary file 8 [file Image_1.jpeg]

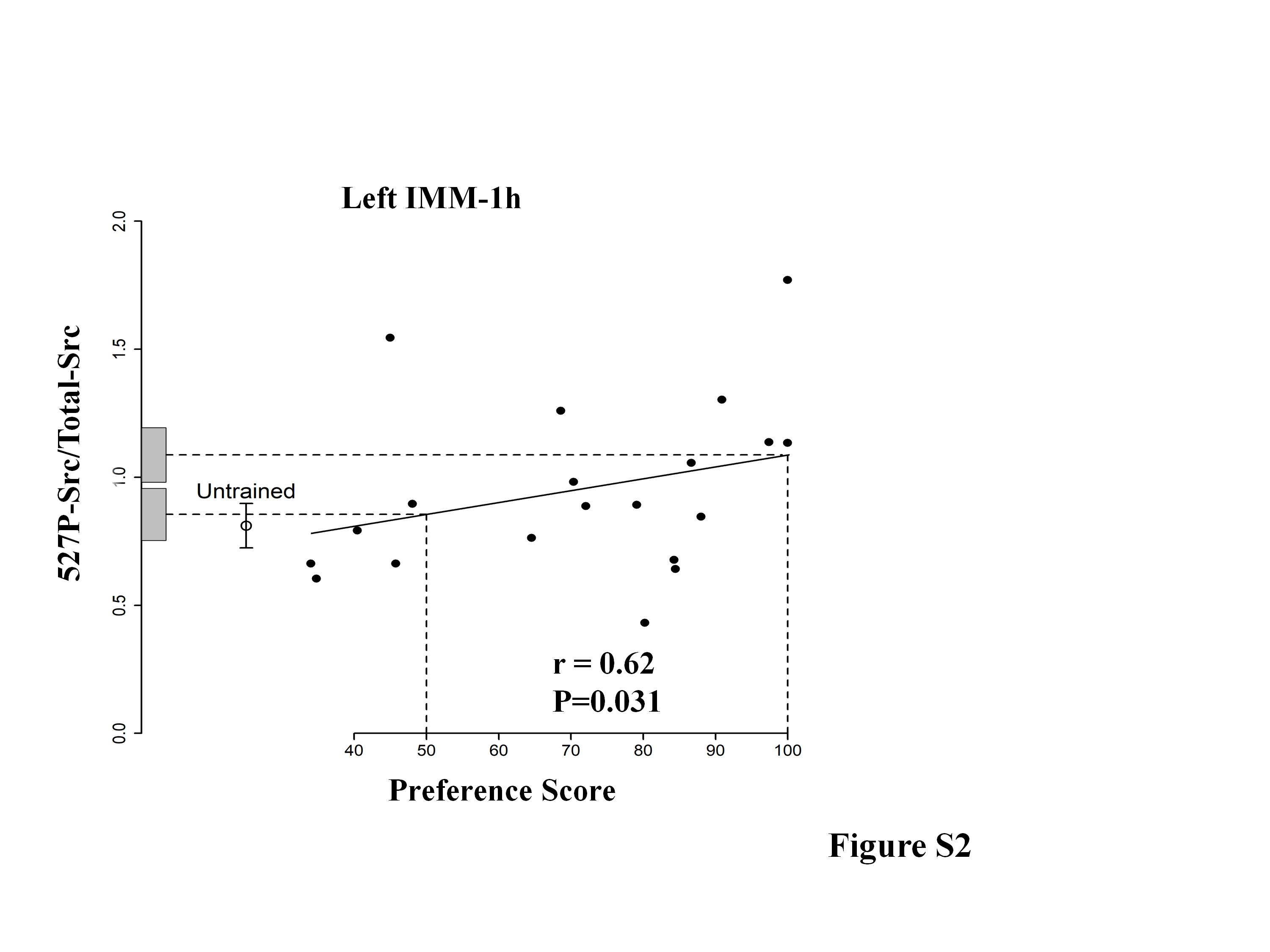

Supplement: Supplementary file 9 [file Image_2.jpeg]
